# Transcriptome meta-analysis reveals differences of immune profile between eutopic endometrium from stage I-II and III-IV endometriosis independently of hormonal milieu

**DOI:** 10.1038/s41598-019-57207-y

**Published:** 2020-01-15

**Authors:** Omero Benedicto Poli-Neto, Juliana Meola, Julio Cesar Rosa-e-Silva, Daniel Tiezzi

**Affiliations:** Gynecological and Obstetrics Department, Ribeirão Preto Medical School of the University of São Paulo, Bandeirantes Avenue, 3900, 8th floor, University Campus, Ribeirão Preto, SP Zip code: 14049-900 Brazil

**Keywords:** Diagnostic markers, Infertility, Translational research, Immunological surveillance, Microarrays

## Abstract

Eutopic endometrium appears to be crucial for endometriosis development. Despite of the evident importance, data regarding the cellular microenvironment remain unclear. Our objective was to explore the tissue microenvironment heterogeneity, transcripts, and pathways that are enriched in all phases of the menstrual cycle by analysing publicly deposited data derived from whole transcriptome microarrays of eutopic endometria of women with and without endometriosis. A meta-analysis of the transcriptome microarrays was performed using raw data available from a public database. Eligibility criteria included eutopic endometrium samples from women with endometriosis and healthy controls without any pathological condition reported the presence of an adequately reported normal menstrual phase, and samples containing both glandular and stromal components. Raw data were processed using a robust multiarray average method to provide background correction, normalisation, and summarisation. The batch effect was estimated by principal variant component analysis and removed using an empirical Bayes method. Cellular tissue heterogeneity was inferred using the xCell package. Differentially expressed genes were identified based on a 5% adjusted p value and a 2.0-fold change. Pathways were identified by functional enrichment based on the Molecular Signatures Database, a p value of < 5%, and an FDR q value of ≤ 25%. Genes that were more frequently found in pathways were identified using leading edge analysis. In a manner independent of cycle phase, the subpopulations of activated dendritic cells, CD4 T effector memory phenotype cells, eosinophils, macrophages M1, and natural killer T cells (NKT) were all higher in stage I-II endometriosis compared to those in healthy controls. The subpopulations of M2 macrophages and natural killer T cells were elevated in eutopic endometriums from women with stage III-IV endometriosis, and smooth muscle cells were always more prevalent in healthy eutopic endometriums. Among the differently expressed genes, *FOS, FOSB, JUNB*, and *EGR1* were the most frequently mapped within the interaction networks, and this was independent of stage and cycle phase. The enriched pathways were directly related to immune surveillance, stem cell self-renewal, and epithelial mesenchymal transition. PI3K AKT mTOR, TGF signalling, and interferon alpha/gamma responses were enriched exclusively in stage III-IV endometriosis. The cellular microenvironments and immune cell profiles were different between eutopic endometriums from women with stage I-II and stage III-IV endometriosis, and these differences were independent of the hormonal milieu. Specifically, a pro-inflammatory profile was predominant in stage I-II endometriosis, and M1-M2 polarization into eutopic endometrium may be crucial for the progression of the disease. The higher prevalence of NKT cells in eutopic endometriums from women with endometriosis that was independent of cycle phase or staging suggested a sustained stress and/or damage to these eutopic endometriums. Based on this, the results of this meta-analysis are important for identifying challenges and opportunities for future research.

## Introduction

Endometriosis is a common disease that affects approximately 5–10% of women at reproductive age; however, the actual prevalence of this disease is difficult to determine, as it varies considerably depending on the population studied. It is found in up to 7% of asymptomatic women subjected to tubal sterilization. Additionally, this disease was identified in 50% of adolescents experiencing difficulty in-controlling dysmenorrhea, in 5%–24% of women with persistent acyclic pain, and in 10%–40% of women suffering from infertility^1,2^. This disease is characterized by the presence of endometrial tissue outside of the uterine cavity, and this most frequent location for this tissue is the pelvis. Endometriosis can affect all organs surrounding the uterus, but it primarily affects the ovaries, sacrouterine ligaments, and pelvic peritoneum^3^. In women, this disease results in a direct negative social and psychological impact on quality of life^4^, and it is also associated with a significant economic cost of US$ 50 billion per year in the USA^5,6^.

Endometriosis exhibits a hereditary component, as it is associated with a familial predisposition that is polygenic and multifactorial; however, this disease is not passed through a simple Mendelian mechanism^7^. The theory of ectopic dissemination of endometrial cells through retrograde menses is still widely accepted as the most important explanation for endometriosis development^8^, but this does not explain all of the nuances of this disease^9^. Retrograde menstruation alone is not solely responsible for the development of endometriosis as this type of menstruation also occurs in most healthy women^10^. At least two additional pivotal mechanisms appear to be fundamental for disease development, and these include immune system dysfunction and genetic susceptibility^11^. Despite these uncertainties, a reasonable amount of information available within the literature indicates that the eutopic endometrium in women with endometriosis is different from that of healthy women. Specifically, the endometria from women with endometriosis exhibit structural changes, the presence of nerve fibres, angiogenesis, receptivity, oestrogen biosynthesis, and progesterone resistance^12^. It is unknown, however, if these alterations are the cause or a consequence of endometriosis

Recent modernization of molecular biology techniques over the last few decades has provided important information that furthered the understanding of several aspects of disease biology^13^, including endometriosis. The analysis of global gene expression, for example, has become relatively accessible, and it has proven useful for determining associations among genomic and phenotypic profiles of various conditions. The two established techniques of transcriptome analysis include the microarray and RNA sequencing. Microarray analysis quantifies the expression of a preselected number of probes/genes determined by certain platforms, while RNA sequencing incorporates high-throughput sequencing to identify all expressed sequences^14^. Despite their strengths and limitations, both methods are reproducible^15^, and because of this, microarray techniques provide researchers with fast, cheap and reproducible results when studying known genes. A number of studies using this technology have identified candidate genes involved in endometriosis pathogenesis^16–19^; however, reduced sample size, sample heterogeneity, inter-individual biological variability, and technical variability (known as batch effects) are some of the potential primary confounders in these studies^20^. Another limitation is the criteria used to interpret the results of these studies. For example, fold change, p-value cut-offs^21^, and ranking metrics for gene set enrichment analysis^22^ can all significantly alter microarray interpretations. These limitations can be at least partially addressed by microarray meta-analysis. This method combines many studies and improves sub-optimal designs. Consequently, it optimizes the power of a given analysis (low false non-discovery and discovery rates) and recognises distinct biological characteristics and phenotypes^23^. Recently developed bioinformatics tools has also provided researchers with the possibility of investigating the heterogeneity of the tissue microenvironment. Although differences in macrophages^24,25^ and dendritic cell^26^ populations have been previously identified upon immunohistochemical analysis of eutopic endometria from women with and without endometriosis, this topic has not being approached in context of the transcriptome. Exploring transcriptome data using a meta-analytical approach can provide the scientific community with substantial, integrated, and confirmatory information regarding the tissue microenvironment and the genes and pathways underlying endometriosis. The results of these studies will also aid researchers in the design of future studies. Just as meta-analyses are important for providing robust evidence from clinical studies, they are also important for ‘omics’ investigations. Identification and confirmation of endometrial markers and processes that is achieved through these studies can provide a basis for developing more secure and less invasive diagnosis and targeted treatment for women suffering from endometriosis.

Here, we describe a meta-analysis of whole transcriptome microarrays from eutopic endometria of women with and without endometriosis. We explored the potential of this method to predict tissue microenvironment heterogeneity. Additionally, the rigorous selection of healthy controls and the use of menstrual phase identification enabled us to identify the most important cell types, transcripts, and pathways that are enriched in eutopic endometria from women with and without endometriosis.

## Material and Methods

We conducted a meta-analysis by combining multiple microarray datasets from samples of eutopic endometria obtained from childbearing women. We performed a search in two public databases for the raw microarray data, and these databases included Array Express (http://www.ebi.ac.uk/arrayexpress/) from the European Bioinformatics Institute (EBI), and Gene Expression Omnibus (GEO) repository (http://ncbi.nlm.nih.gov/geo/) from the National Centre for Biotechnology Information (NCBI). The search was performed using the following keywords: “endometriosis” or “endometrium” or “uterus” and “GPL570” (the platform accession name for high-density oligonucleotide microarray Affymetrix Human Genome U133 Plus 2. Array - HG-U133 Plus 2) (Affymetrix, Santa Clara, CA). We chose the Affymetrix GeneChip arrays platform based on the knowledge that it is the most actualized and dominant product on the market and is used worldwide. Additionally, although cross-platform normalization is possible, we may include critical batch effects that, when removed, may minimize the significance of the biological effect. Initially, a search of the GEO DataSets identified 1487 results for Homo sapiens, and among these results we found 15 datasets. In Array Express, we identified 2 experiments. Only studies that publishe raw data were considered eligible for inclusion in our meta-analysis. We included eutopic endometrium samples from women with endometriosis and healthy controls without any other pathological condition reported, where the menstrual phase was adequately reported for both groups and the samples contained both glandular and stromal components. We chose these criteria due to the significant molecular phenotypic differences presented by the eutopic endometrium in the various phases of the cell cycle^17,27^ and the importance of the microenvironment in the pathophysiology of the disease^28^. Data were obtained from GSE4888^27^, GSE6364^17^, GSE7305^29^ and GSE51981^30^. GSE7307 was not selected owing to its lack of information regarding if women were childbearing or postmenopausal, and GSE29981 was not selected because it included only glandular component analysed after laser capture microdissection. For analysis, we divided the endometriosis samples in two groups that included stage I-II and stage III-IV groups. We also focused on common alterations that occur throughout the menstrual phases.

All computational analyses were performed in the R environment. The CEL format files containing the microarray experimental data were downloaded and processed using the robust multiarray average method (RMA) to allow for background correction, normalisation and summarisation^31,32^. After pre-processing, the probe expression level was collapsed to the corresponding gene using the highest value (maximum) of expression in each sample.

The presence of numerous cell types within samples can influence the quality of microarrays interpretation and can consequently affect biological conclusions^33^. Based on this, we determined the tissue cellular heterogeneity using the xCell package^34^, a gene-based marker method capable of differentiating among 64 immune and stromal cell types, and CIBERSORT^35,36^, a deconvolution-based approach that can be used to differentiate among 22 immune cell subsets. The use of xCell allowed for comparability between samples, while CIBERSORT generates a relative cell fraction score, that allows only an intra-sample comparison, although it has been extended to an ‘absolute mode’ (beta version) which provides a score that can be compared between samples. xCell initially computes individual cell scores (an arbitrary unit), and it ultimately grouped these values into immune and stromal scores that comprise the microenvironment score.

**Figure 1:**
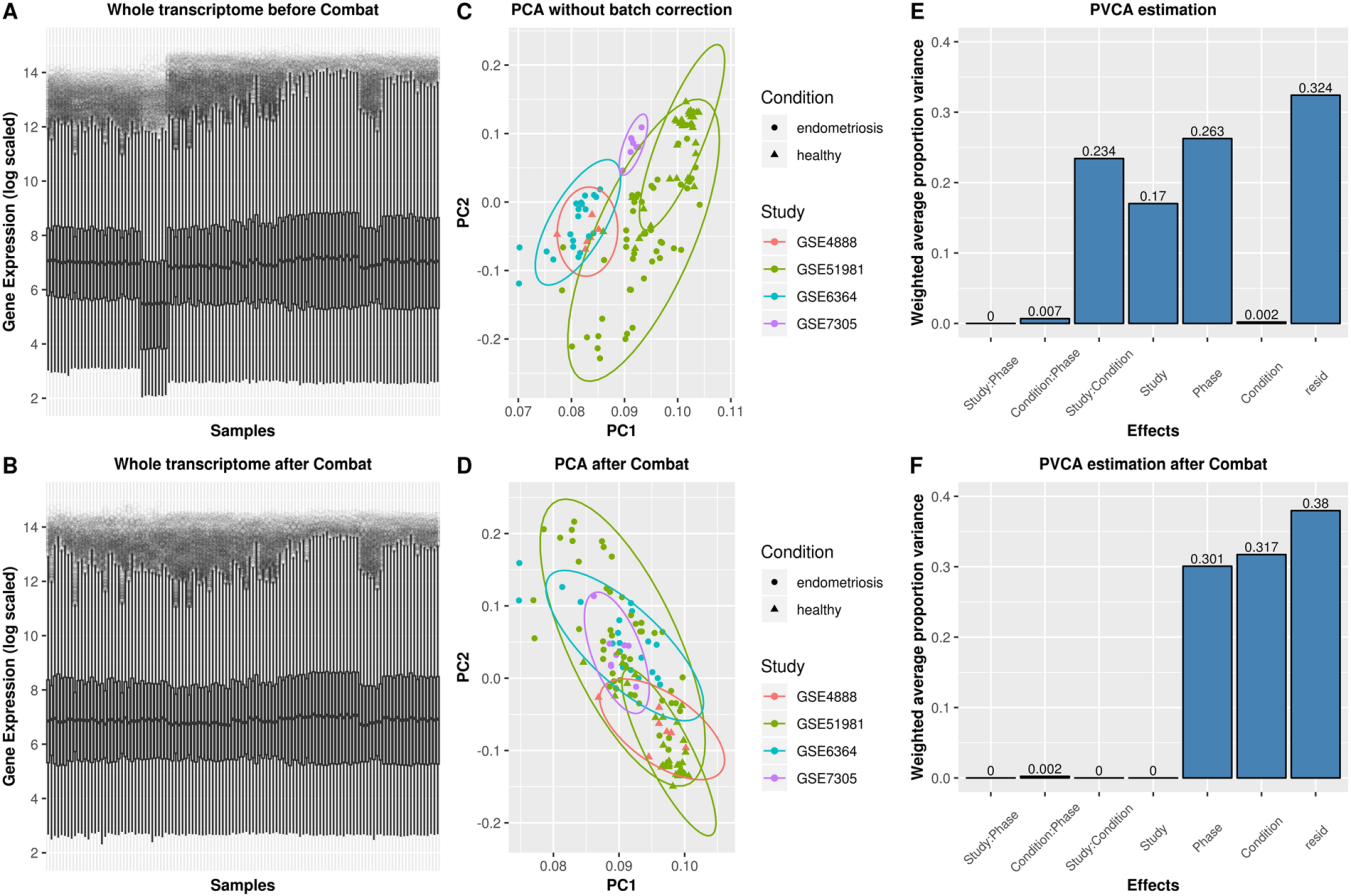
Gene expression after combining data from datasets, identification and removal of the batch effect. Notes: Boxplots show intensity of the log2-transformed gene expression before (**A**) and after (**B**) batch effect removal. Scatterplots show PCA analysis of normalized gene expression data before (**C**) and after (**D**) batch effect removal by Combat; ellipse underlying assumptions about the distribution of the data was drawn considering a multivariate t-distribution and a confidence level of 0.95. Bar charts show the proportion of batch effect by PVCA estimation from possible sources before (**E**) and after (**F**) batch correction. ComBat with parametric adjustment was used to remove the estimated batch effect.

After generating these initial data, we unified the databases and estimated the potential non-biological experimental variation (batch effect) derived from combining multiple datasets by principal variant component analysis (PVCA)^37^, which is a hybrid approach that incorporates principal component analysis (PCA) and variance component analysis (VCA). After the identification, the batch effect was removed by ComBat, an empirical Bayes method^38^ (Fig. 1).

Prior to analysing the differentially expressed genes, we performed a non-specific filtering where we imposed only one requirement, where the estimated intensity must be higher than 100 fluorescence units in at least 25% of the samples. Genes that passed the filter were referred to as expressed genes. Then, we plotted a heatmap to visualize the hierarchical unsupervised clustering using the Ward D method, and the distance between measures were based on Euclidean distance. We estimated the ideal number of clusters using the elbow, silhouette and gap statistical methods^39,40^.

For identification of differentially expressed genes (DEG), we use the Limma package^41^. First, we assessed the empirical array quality weights^42^,as these values increase statistical power to detect true differential expression without increasing the false discovery rate. All comparisons were performed between menstrual phase-specified for endometriosis samples and healthy samples. Initially, to select the most important gene markers, we set the cut-offs at 5% for adjusted p value and at 2.0 for fold change. We also used the STRING database to summarize the network of predicted associations for the group of proteins represented by the most significantly DEG, where the high score was set at 700^43^.

As statistically significant gene expressions are not necessarily biologically meaningful for a given biological condition, we performed functional enrichment using all genes pre-ranked by signal-to-noise ranking metric without filtering. The analysis was performed using GSEA Software 3.0^44^ and the Molecular Signatures Database (MSigDB 6.2 released), which possesses a wide collection of annotated gene sets^45^. We recognize that there are other excellent tools for enrichment analysis^46,47^, but we believe that the method employed could overcome two common limitations in this type of analysis. Specifically, this approach allowed us to 1) include the complete list of genes in the analysis and thus avoid the use of arbitrary thresholds for gene selection and to 2) identify key pathways in a concise, non-redundant manner to facilitate first interpretation of results. Then, we initially applied GSEA to the hallmark gene sets to summarise well-defined biological conditions of the original founder sets to reduce both variation and redundancy^48^ from numerous pathway/gene sets databases such as BioCarta^49^, Kyoto Encyclopaedia Genes Genomes^50^, Reactome^51^ Gene Ontology^52^, miRBase^53^, Transfac^54^, MYC Target Gene^55^, Pathway Interaction Database^56^, and others^57–63^. We used parameters that included 1000 permutations, weighted enrichment statistics (p value = 1), and the exclusion of gene sets with size larger than 500 and smaller than 15 genes. For interpretation, a p value of < 5% and a false discovery rate (FDR) q value of ≤ 25% was considered significant as suggested by authors. The enrichment score reflected the degree to which the genes in a gene set are overrepresented. Positive and negative signal in the ES indicated correlation with the gene set enrichment at the top or the bottom of the ranked list, that is, genes up or down regulated. The ES were adjusted for variation in gene set size and, then, represented by normalized enrichment scores (NES). More details can be obtained by consulting documentation in http://www.gsea-msigdb.org/gsea/index.jsp.

To determine which genes exert the highest impact on the biological process under study (all representative hallmark pathways in each menstrual phase), we performed a leading edge analysis (LEA). The LEA allows us to determine which subsets (referred to as the leading edge subset) of genes contributed the most to the gene sets enrichment signal. This analysis included all genes that appeared in the ranked list at or before the point at which the running sum reached its maximum deviation from zero. It is likely that a gene present in many leading edge subsets is more interesting ore more important than genes that appear in only a few subsets. The analysis was also performed using GSEA Software 3.0.

## Results

**Figure 2:**
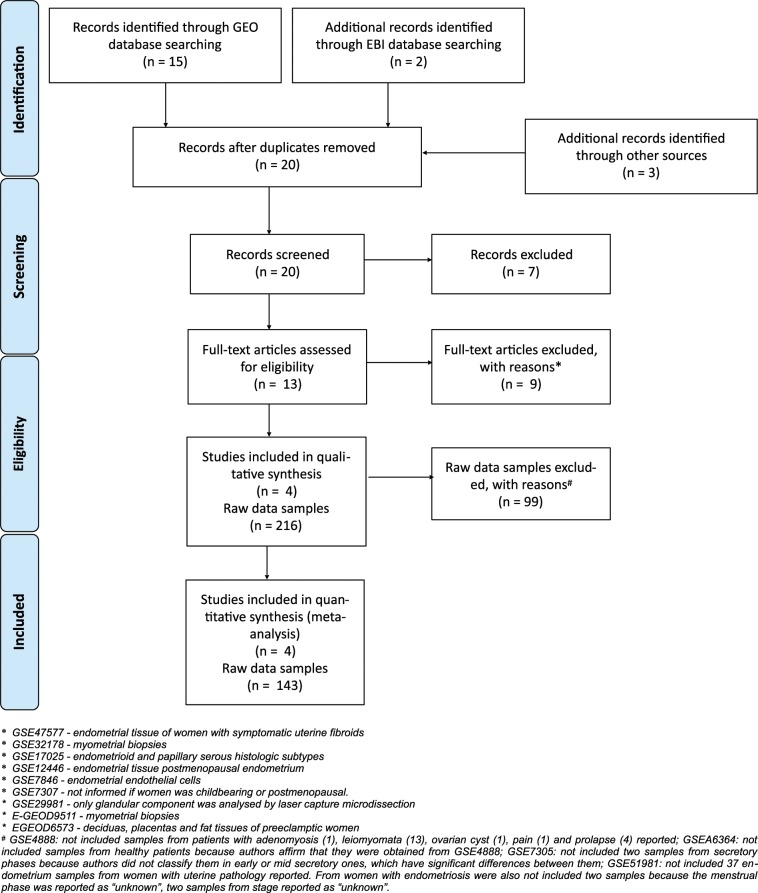
Flow diagram showing the process to obtain information for the meta-analysis.

Our dataset selection strategy is presented in a PRISMA flowchart (Fig. 2). Our casuistic was composed of 41 samples from healthy women and 102 samples from women with endometriosis (26 stage I-II, 76 stage III-IV) (Table 1).

**Table 1:** Datasets and samples selected by searching in Pubmed and GEO repository.

GEO series/Study	Reference	Samples	Eutopic endometrium	Cycle phases
GSE4888	30	7	Healthy	Proliferative (3) Mid secretory (4)
GSE6364	20	21	Endometriosis	Proliferative (6) Early secretory (6) Mid secretory (9)
GSE7305	32	8	Endometriosis	Proliferative (8)
GSE51981	33	107	Healthy / Endometriosis	Proliferative (20/28) Early secretory (6/18) Mid secretory (8/27)

**Figure 3:**
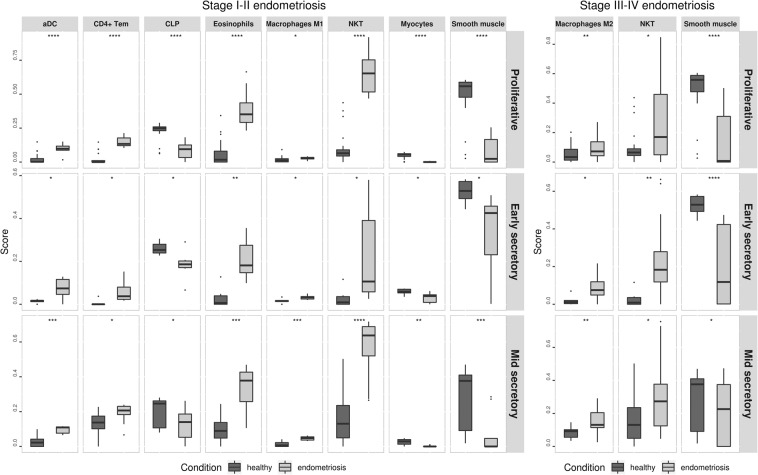
Cell subtypes identified as differently scored in eutopic endometrium samples from stage I-II and stage III-IV endometriosis compared to healthy controls.

Immune scores were higher in samples from women with stage I-II (0.344 ± 0.031; p = 0.006), but not in stage III-IV (0.284 ± 0.024; p = 0.487) endometriosis when compared to the scores of healthy individuals (0.261 ± 0.030). Stroma scores were not significantly different between stage I-II (0.084 ± 0.014; p = 0.513) or stage III-IV (0.063 ± 0.008; p = 0.087) endometriosiswhen compared to those of healthy women (0.075 ± 0.004). Throughout each cycle phase, subpopulations of activated dendritic cells (aDC), CD4 T effector memory phenotype cells (CD4 TEM), eosinophils, macrophages M1, natural killer T cells (NKT), and myocites were predominant in stage I-II endometriosis compared to levels observed in healthy controls, while common lymphoid progenitors (CLP) were predominant in these lasts. Additionally, subpopulations of M2 macrophages and natural killer T cells (NKT) were elevated in eutopic endometria of women with stage III-IV endometriosis, and smooth muscle cells were always more prevalent in healthy eutopic endometrium (Fig. 3). These details are presented in the supplementary datasets (Datasets 1A–1F). CIBERSORT did not identify consistent differences in immune heterogeneity between samples from endometriosis and healthy eutopic endometrium, and less than half of the samples reached a recommended p-value threshold of < 0.05 for the global deconvolution.

**Figure 4:**
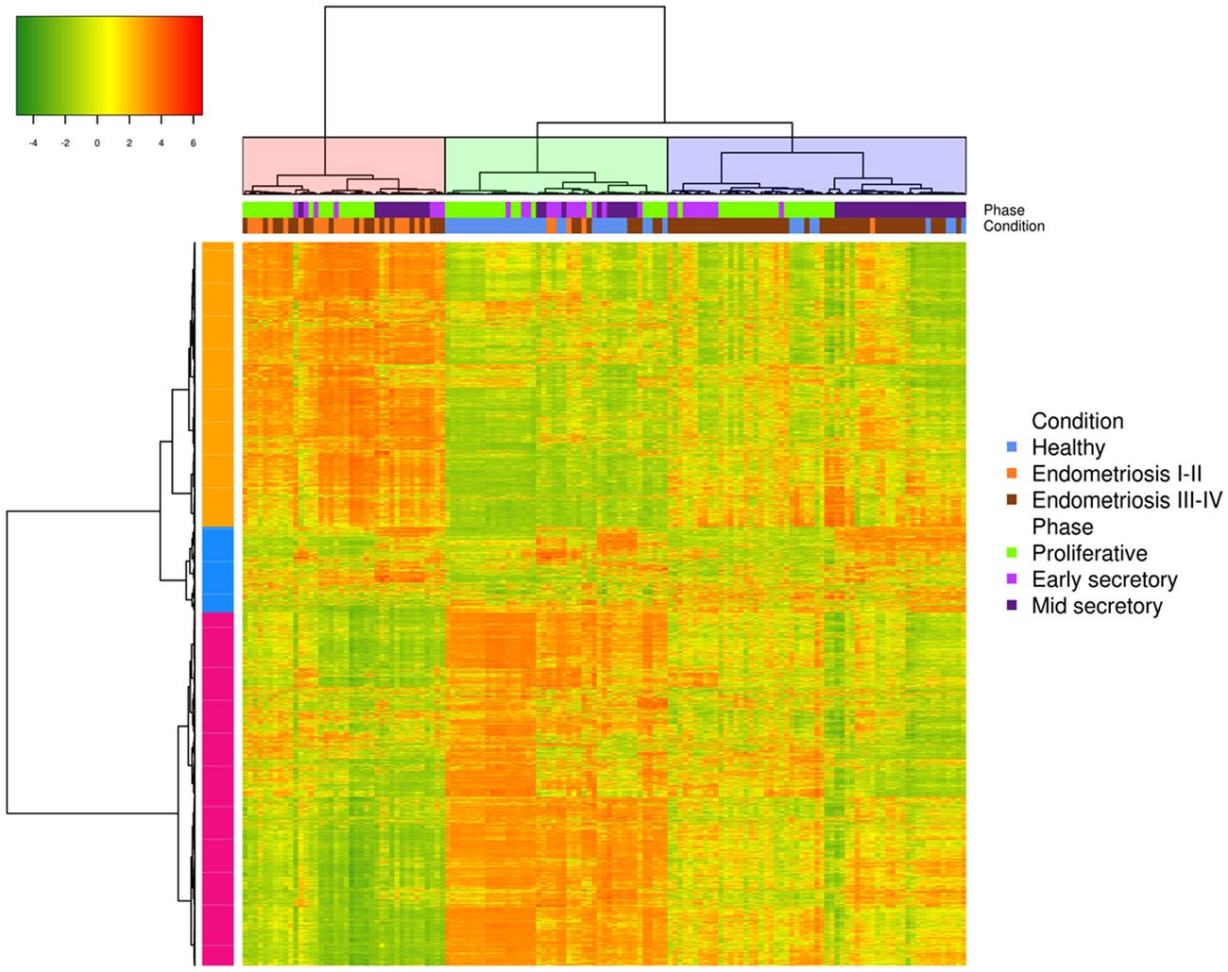
Heatmap and hierarchical clustering of gene expression levels in eutopic endometrium of women with endometriosis and healthy controls. Notes: Rows represent genes, and colunms represent samples (healthy controls, stage I-II, and stage III-IV are predominantly grouped in clusters green, pink and violet, respectively).

From the 20,192 collapsed genes, 12,460 genes were selected after filtering for gene expression analysis. The unsupervised hierarchical cluster heat map revealed three main clusters that segregated eutopic endometrium samples from those of healthy women (“green”) and women suffering from stage I-II endometriosis (“pink”) or stage III-IV endometriosis women (“violet”) (Fig. 4). These findings were reinforced by both methods that were used for cluster number selection.

**Figure 5:**
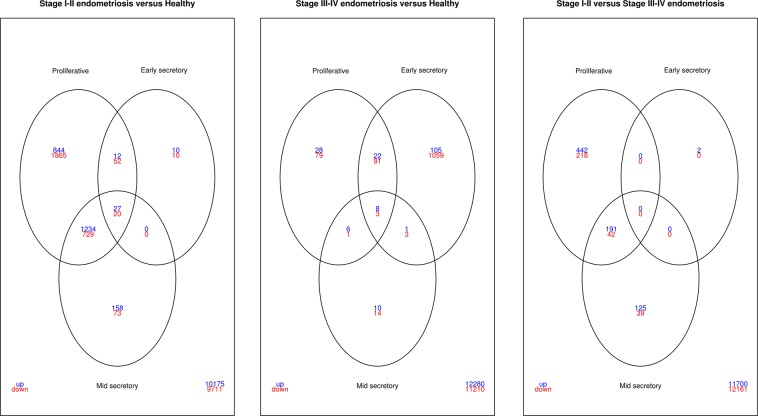
Venn diagrams of differentially expressed genes (DEGs) (up/down) between the conditions throughout menstrual phase and endometriosis staging.

**Figure 6:**
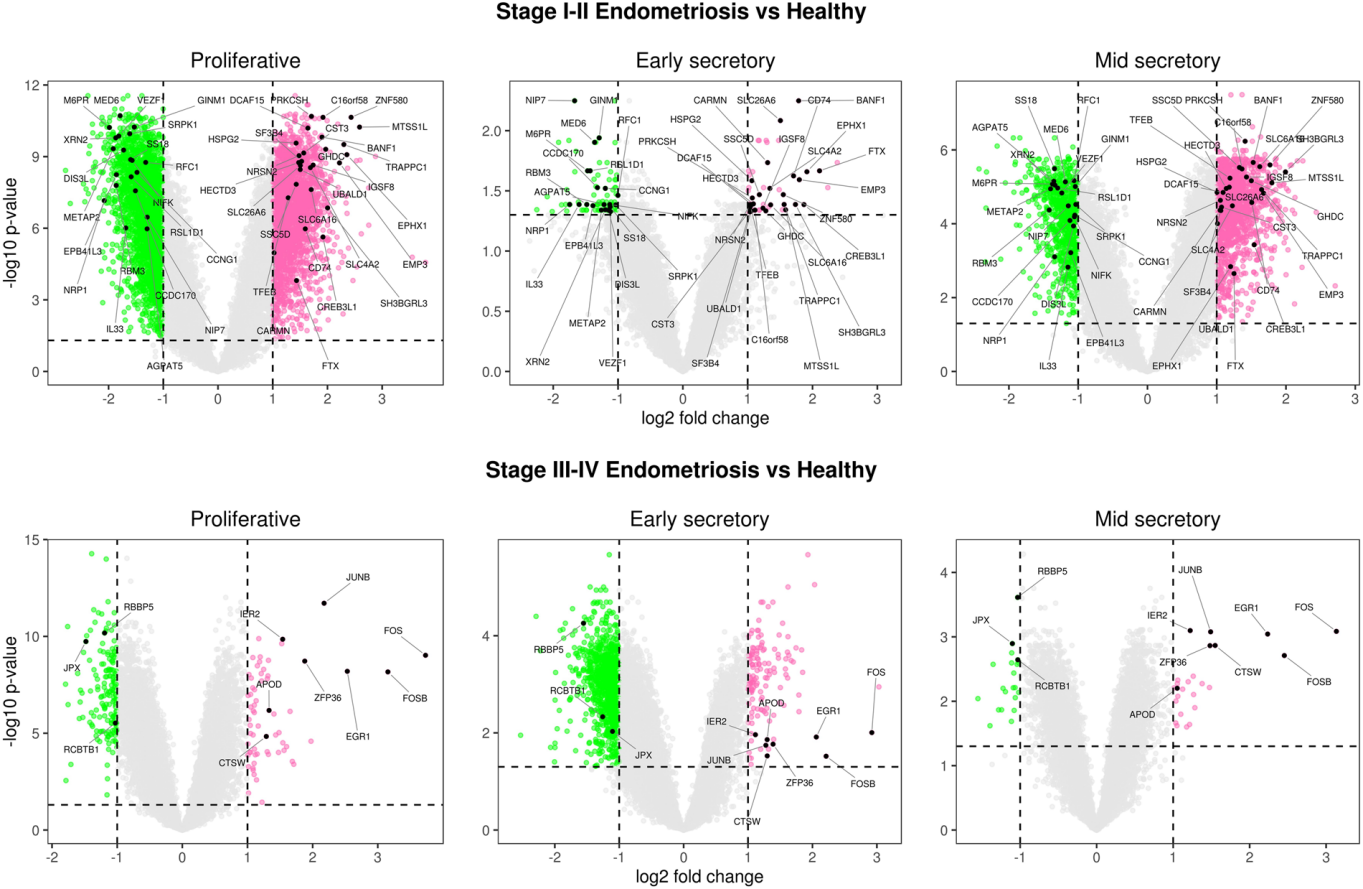
Volcano plots showing gene expression in each menstrual phase and endometriosis staging. Notes: Horizontal and vertical dashed lines represent, respectively, adjusted p value (0.05) and fold change (2.0). Filled black dots represent DEGs identified in both cycle phases, proliferative, early and mid secretory, considering adjusted p < 0.05 and FC >2.0 (logFC >1.0).

The empirical array quality weights were heterogeneous, and they varied from 0.22 to 3.07. These weights were subsequently used in the linear model analysis. The number of up/down genes that were differently expressed between endometriosis and healthy control and between stage I-II and stage III-IV endometriosis are presented in Fig. 5, in combination with the overlap between DEGs according to cycle phases. Overlapped genes are highlighted in the volcano plot (Fig. 6). The full DEGs list according to menstrual phase is presented in the supplementary datasets (Datasets 2A–2F). Gene cluster interaction networks were constructed for the proliferative, early-, and mid-secretory phases, and these networks can be accessed, respectively, by the following links: http://version10.string-db.org/10/p/1667483704, http://version10.string-db.org/10/p/9942483705, http://version10.string-db.org/10/p/4380483706 (for stage I-II endometriosis compared to healthy controls); and in: http://version10.string-db.org/10/p/4168483707, http://version10.string-db.org/10/p/7463483708, http://version10.string-db.org/10/p/8498483709 (for stage III-IV endometriosis compared to healthy controls). The coloured halo surrounding the bubbles represents the level of gene expression. Red-tagged genes are up-regulated, and green-tagged genes are down-regulated. By accessing the link the reader can change other parameters of the analysis and can observe characteristics such as the type of interaction and the enriched pathways. We observed that the most frequent interactions occurred among the *FOS*, *FOSB*, *JUNB*, and *EGR1* in a manner that pratically independent of stage and cycle phase. Additionally, the interaction scores among these proteins are also high (*FOS* × *JUNB* = 0.999, *FOSB* × *JUNB* = 0.998, *FOS* × *EGR1* = 0.984, *FOSB* × *EGR1* = 0.776).

**Figure 7:**
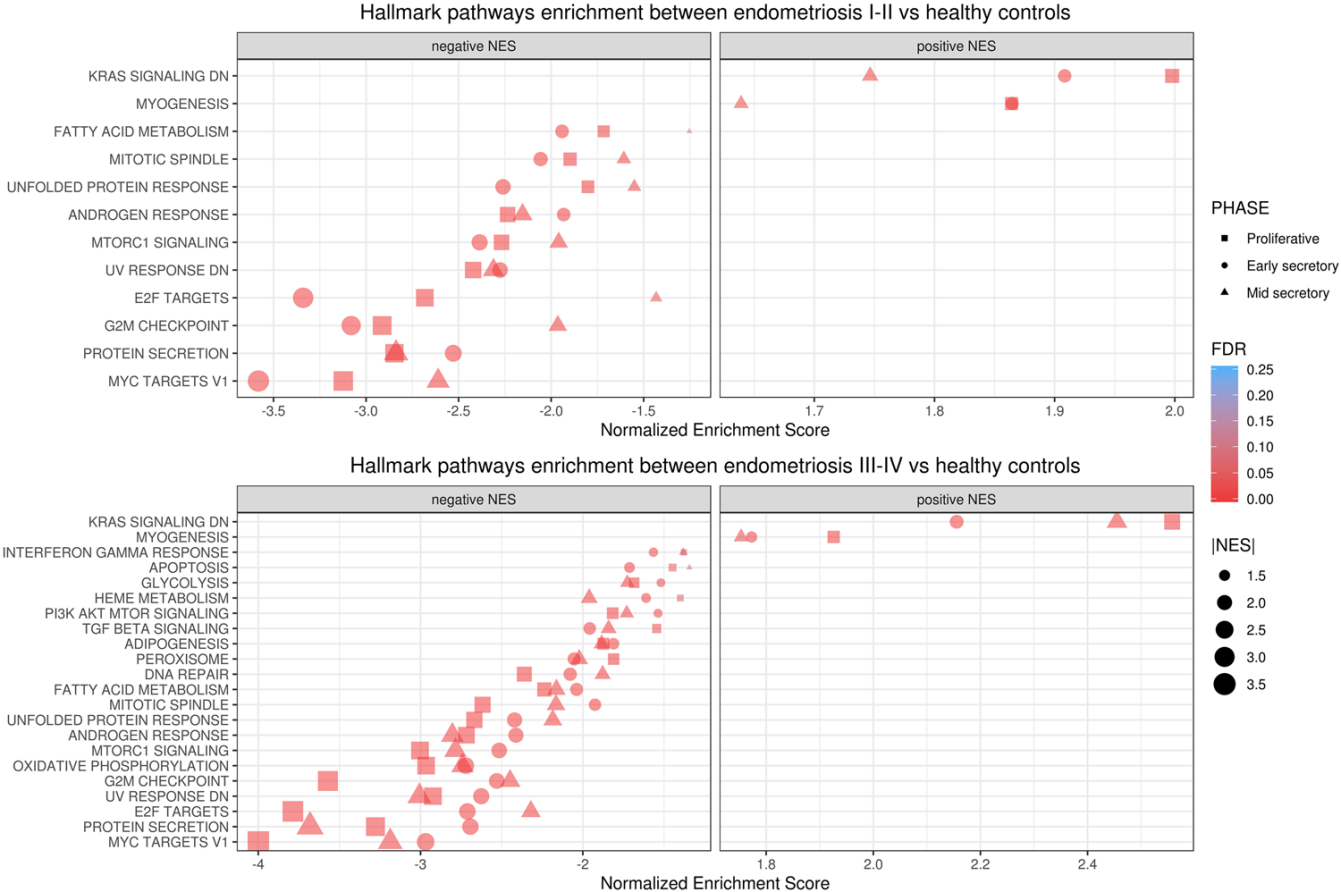
Graphic representation of Hallmark pathways commonly identified in all cycle phases according to endometriosis staging. Notes: FDR = false discovery rate; NES = normalized enrichment score.

The hallmark gene sets that were enriched throughout the cycle phases are represented in the Fig. 7, and they are arranged according to endometriosis staging. Distinct pathways that existed between stage I-II and stage III-IV endometriosis included: adipogenesis, PI3K AKT mTOR signalling, peroxisome, glycolysis, TGF beta signalling, heme metabolism, and interferon gamma response. The full list of pathways enriched in the hallmark dataset according to each menstrual phase is presented in the supplementary datasets (Datasets 3A–3F). For stage I-II endometriosis that was compared to healthy samples, we used LEA to identify 1,338, 1,264, and 1,162 core genes, respectively, in proliferative, early, and mid secretory cycle phases. For stage III-IV endometriosis, LEA identified 1,522, 1,455, and 1,303 core genes, respectively, in these same cycle phases. Table 2 indicates the proportion of core genes that are involved in two or more leading edge subsets, specifically, genes participating consistently in more than one enriched pathway. The gene *NOLC1* is the most frequently found in multiple leading edge subsets derived from enriched pathways throughout the menstrual phases, in stage I-II endometriosis. In contrast, *CDKN1B*, *DLD*, *ELOVL5*, *H2AFZ*, *IDI1*, *ME1*, *MTHFD2*, *NOLC1*, and *SOD1* were commonly present in stage III-IV endometriosis.

**Table 2:** Number of core genes participating in more than one leading edge subsets by each menstrual phase considering endometriosis versus healthy controls.

Endometriosis staging	Leading edge subsets that a gene participates	Menstrual phase		
		Proliferative	Early secretory	Mid secretory
I-II stage		(n = 1338)	(n = 1264)	(n = 1162)
	> = 2, n[%]	242 [18.1]	307 [24.3]	168 [14.5]
	> = 3, n [%]	43 [3.2]	88 [7.0]	29 [2.5]
	> = 4, n [%]	8 [0.6]	18 [1.4]	1 [0.1]
	> = 5, n [%]	0 [0.0]	7 [0.6]	0 [0.0]
	> = 6, n [%]	0 [0.0]	3 [0.2]	0 [0.0]
III-IV stage		(n = 1522)	(n = 1455)	(n = 1303)
	> = 2, n[%]	362 [23.8]	380 [26.1]	337 [25.9]
	> = 3, n [%]	97 [6.4]	92 [6.3]	82 [6.3]
	> = 4, n [%]	17 [1.1]	13 [0.9]	15 [1.2]
	> = 5, n [%]	7 [0.5]	5 [0.3]	6 [0.5]
	> = 6, n [%]	2 [0.1]	0 [0.0]	3 [0.2]

The leading-edge subset can be interpreted as the core group of genes that accounts for the gene set’s enrichment signal.

## Discussion

### Microenvironment

To the best of our knowledge, this meta-analysis is the first study to use whole transcriptome analysis to digitally portray the microenvironment landscape of eutopic endometria from women with and without endometriosis through the use of in silico analyses. We identified significant differences in the scores of various immune and stromal cell types throughout all menstrual cycle phases, suggesting the existence of an intrinsic eutopic endometrium condition that was independent of the hormonal milieu. Within the eutopic endometria obtained from women with stage I-II endometriosis there was a predominance of several cell subtypes (aDC, CD4 T cells, CD4 TEM, eosinophils, macrophages M1, NKT); however, this difference was less significant in the endometria obtained from women with stage III-IV endometriosis, which predominantly contained only M2 macrophages and NKT. Additionally, smooth muscle cells were always more prevalent in healthy subjects than they were in individuals suffering from endometriosis.

It is well known that the relationship between endometriosis and the immune system is intimate^64–66^. Macrophages, for example, have long been known to act as important cells within the normal eutopic endometrium^67^ and in the etiopathogenesis of endometriosis, where they are potentially responsible for survival, neovascularization, the growth of ectopic lesions^68^, and the formation of endometriomas^69^. These cells respond to signals from the microenvironment and adopt different functional programs in a process named polarization. There are roughly two populations of these cells, including the classically activated M1 and alternatively activated M2 macrophages. The first is classically activated by lipopolysaccharide (LPS) and interferon, and it possesses pro-inflammatory and bactericidal functions during acute infections. M2, in contrast, can be polarized by different types of stimuli and these cells play potential roles in immunoregulatory and anti-inflammatory processes such as wound healing, tissue repair, angiogenesis, and immune system activation^70^. These cells are also associated with the promotion of tumour growth^71^ and the later stages of infectious diseases^72^. In animal models, alternatively activated M2 macrophages appear to be required for the development of ectopic lesions^73,74^. In humans, the distribution of these cells within the eutopic endometrium remains unclear. Cominelli *et al*. claimed that M2 phenotype were more abundant than were M1 macrophages^75^. In contrast, Takebayashi *et al*. used paraffin-embedded specimens and immunostaining to reveal a lower ratio of M2 macrophages in the endometriosis group^25^. It is possible that the presence of multiple pathological diagnoses in the uterus and the use of CD68 as pan-macrophage markers may have contributed to these different findings. Today, it is also known that CD68 immunoreactivity is detected in diverse cell types, including dendritic cells, NK cell, basophils, fibroblasts, endothelial cells, and even in M1 macrophages^76^. In fact, there are no ideal surface markers that can distinguish between M1 and M2 macrophages^77^. Based on this, we believe that a tool based on a gene signature-based method that is learned from pure cell types from various sources such as xCell is more reliable, as it has been demonstrated that this tool exhibits the greatest ability to identify macrophages in biological samples^78^. Our meta-analysis indicates that M1 macrophages are more prevalent in stage I-II, while M2 macrophages are more prevalent in stage III-IV endometriosis. These data together with the presence of other typically pro-inflammatory cell subtypes suggest that the endometrial environment in early disease could even be more pro-inflammatory than in late disease. Our study does not, however, allow us to make definitive conclusions regarding macrophage activity. An LPS-inducible phenotype is a typical feature of M1 macrophages; however, M2 macrophages can also be LPS-inducible and can exhibit pro-inflammatory behaviour^76^. This macrophage activity is partially modulated by Toll-like receptors (TLRs)^79^, which play an important role in the relationship between innate immunity and bacterial endotoxin and in endometriosis^80^. Additionally, the activation of TLRs regulates stem cell proliferation and differentiation, guaranteeing a multipotent profile^81^ and interfering with the immunosuppressive role of endometrial stem cells in endometriotic tissue^82^ and the modulation of the innate immune system^83–85^. The activation of TLRs in non-cancer stem cells also significantly reduces the expression of *RBBP5*^86^ and could justify the abnormally low levels of this transcript that were observed in the endometria of women suffering from endometriosis. The presence of high numbers of M2 macrophages in the endometrium could be used as a marker of endometriosis^87,88^.

Natural killer T cells, in turn, are a subset of T cells that share structural and functional characteristics with both T lymphocytes and natural killer cells. They are regarded as sentinels of tissue integrity, where they recognize local tissue stress and damage. The majority of these cells recognize glycolipids that are presented by the CD1d antigen presenting molecule. This action is typical of an innate immune response against micro-organisms and of as well as tumour immunity processes such as immunosurveillance^89^. Previous studies have observed reduced cytotoxic function of peripheral and peritoneal natural killer cells in women with endometriosis^90^; however, information regarding NKT cells in the context of eutopic and ectopic endometria remains scarce^91,92^ and requires further study. In fact, it has been demonstrated that these cells are more prevalent in the peripheral blood of women with endometriosis compared to the levels in healthy women^93^. Through their linkage to diverse immune effector functions^94^, these cells may play a key role in the immunopathogenesis of the endometriosis. Functionally, NKT cells can drive the immune response toward inflammation or toward tolerance. In the early stages of cancer development, NKT cells may promote the maturation of dendritic cells and even assume a T helper 1 phenotype to induce an effective antitumor response. In contrast, when chronically stimulated, these cells can become anergic and switch to a T helper 2/T regulatory profile, to promote M2 macrophage polarization and facilitate immune escape and consequent tumour progression^95^. Based on our findings regarding NKT cells and M2 macrophages and on the evidence that endometriosis is a chronic inflammatory disease, at least the later stages, we hypothesised that the anergy of NKT cells may promote M2 macrophage polarization in the eutopic endometrium. This phenotype could contribute by ensuring production of extracellular matrix, angiogenesis, and immune escape, which are all pivotal elements in the development, maintenance, or even progression of endometriosis from earlier to later stages. Our data reinforce the idea that the search for immunomodulators is proving to be promising for the treatment of the endometriosis, despite the fact that there have been no definitive successes to date^96^. New advances in the modulation of the M1-M2 macrophage polarization^97,98^ and in targeting NKT cells^99^ will provide a foundation for more effective treatment of this disease.

We have also identified more abundant level of activated dendritic cells in the eutopic endometria from women with stage I-II endometriosis when compared with the healthy controls. As these cells are critical players in the deflagration and development of immune response^100^, it is plausible to hipothesize that the eutopic endometria of these women can be exposed to a relatively recent aggression. Another interesting finding of our meta-analysis is the identification of a higher prevalence of eosinophils in stage I-II endometriosis samples. The presence of high levels of these cells are associated with chronic endometritis^101^, promotion of the endometrial stromal cell proliferation after infectious insult^102^, the antigen presentation, dendritic cell activation using recognition of pathogen-associated molecular patterns, and macrophage polarization^103^. In addition to our observation of increased levels of defensins, natural components of human innate immune response^104^, in the endometriosis group, our data highlighted the association of endometriosis and the host innate immune response to tissue aggressors such as infectious agents. This is in agreement with a new concept called the “bacterial contamination hypothesis” proposed by Khan *et al*.^105^, which is based on several studies showing an association between endometriosis and endometritis^106–109^ and microbial contamination of the uterine cavity or the contamination of ectopic lesions by various agents^110–112^.

In regard to the higher prevalence of smooth muscle cells within healthy endometria, this finding can explain the higher expression of caldesmon (CALD1), a biomarker of smooth muscle differentiation, in normal endometrium compared to the levels in endometria from women with endometriosis^113^. Considering the method of endometrium sampling that was employed in the original studies included in our meta-analysis, the myocytes likely originated from endometrial-myometrial interface. These cells likely possess asynthetic phenotype in which there is a predominance of organelles in relation to contractile components^114^. They play an important role in regulating the microenvironment, by influencing the proliferation and differentiation of myoblasts^115^ in a manner that may be dependent upon the action of progesterone. We believe that at least two hypotheses should be raised, either individually or together. First, the mammalian target of rapamycin (mTOR) pathway is essential for myoblast differentiation^116^. Given that several pathways involving mTOR are compromised in the endometria of women with endometriosis, it is possible to hypothesize that in the normal endometrium the differentiation is more frequent and a naturally occurring process, which would justify the difference in the quantity of myocytes. Second, considering that myoblast differentiation also depends on progesterone^117^ and that there is greater resistance to the action of progesterone in the endometrium of women with endometriosis^17^ that is cause either by negative modulation induced by inflammation^118^ or by repression promoted by miRNAs^119^, we can also hypothesize that this process is preserved in the healthy endometrium and not in the diseased one, which would also justify the difference.

### Differently expressed genes

Our meta-analysis reinforces the central role of previously reported dysregulated genes (*FOS*, *FOSB*, *EGR1*, and *JUNB*) (30). STRING database analyses revealed high interaction scores among these genes. In parallel, these genes are directly and jointly related to macrophage differentiation and activation^120,121^, and they are also related to the expression profiles of NKT cells^122^. Other dysregulated genes from more advanced endometriosis are also involved in macrophage polarization, an event that we previously hipothesised as potentially important for disease progression.

Both *FOS* and *JUNB* can dimerize and form the activator protein 1 (AP-1) transcription factor that regulates gene expression in response to a wide variety of stimuli, including cytokines, growth factors, tissue stress, and innumerable cellular processes such as differentiation, proliferation, and apoptosis^123^. These proteins can also regulate early growth response protein 1 (*EGR1*)^124^, as its expression is coregulated by *FOS*^125^ and its transcriptional regulation in inflammatory processes depends on *JUN*^126^. High expression of *FOS* has already been reported in the eutopic endometrium of women with endometriosis. Further, the expression of *FOS* was associated with higher peripheral levels of 17ß estradiol and local levels of MMP9 in these women^127^. The *ZFP36* encodes the RNA binding protein tristetraprolin (TTP) that acts as a post-transcriptional regulator of inflammation by binding and destabilizing various cytokines. Khalaj *et al*. previously observed that TTP has the potential to regulate the inflammatory process associated with endometriosis by interacting with tumour necrosis factor alpha (TNF-α), granulocyte macrophage colony stimulating factor (GM-CSF), interleukin 6 (IL-6), cyclooxygenase-2 (COX-2), hypoxia-inducible factor 1alpha (HIF-1α), and interferon gamma (IFN-γ)^128^. *IER2*, in addition to *FOS* and *JUN*, is an immediate early gene that can be induced by proliferation and migration stimuli, and this gene contributes to angiogenesis, cell motility, adhesion^129^, and tumour progression^130^. *APOD* encodes an atypical lipoprotein from the lipocalin family that is expressed in the normal endometrium^131^ and is responsible for the transport of small lipophilic molecules^132^, including sexual steroidal hormones, that, in turn, modulate its translation^133,134^. Overexpression of apoD appears to be a tissue strategy designed to resist oxidative stress and inflammation^135^, and to prevent lipid peroxidation by converting reactive lipid hydroxides into non-reactive lipid hydroxides. *CTSW* encodes cathepsin W, a protein that exhibits a restricted cell distribution^136^ and plays a specific role in regulating the activity of NKT cells^137^ and CD8 cytotoxic T cells^138^ such as TEM cells. Additionally, elevated expression of *CTSW* is associated with favourable prognosis in patients diagnosed with endometrial cancer^139^. *JPX*, a long, non-coding RNA X-inactive specific transcript activator, can also be involved in M1-M2 macrophage polarization^140^.

Additionally, among common DEGs in stage I-II endometriosis, several of them are relative to inflammatory and/or infectious process. *ZNF580* is potentially involved in the modulation of inflammatory process^141^; *DCAF15* is potentially involved in the immune surveillance^142^; *BANF1* is involved in the immunity against integration of foreign DNA and response to DNA damage^143^, and it is required to maintain undifferentiated phenotype of the stem cells^144^; *HECTD3* is associated to the modulation of host defense against infection^145^; *SSC5D*, which is a soluble receptor produced by macrophages, T cells, and epithelial cells from placenta, is upregulated on infection and it has capacity to interact with bacteria^146^; *TEFB* has a role in the autophagy and in the regulation of inflammasome^147^; *CD74* plays a role in the macrophage recruitment, adhesion and migration^148^. Despite the debatable utility of the biomarkers as noninvasive tool to diagnosis endometriosis^149,150^, these differences in transcript levels should be investigated further, at least as a driver to understand its pathophysiology.

### Enrichment analysis

Numerous pathways identified in our study are involved in cell cycle control and immune modulation, including M2 macrophage polarization^151–153^ and NKT maturation/activation^154–156^. Additionally, these pathways are also extensively reported in other processes such as immunosurveillance^157^, stem cell self-renewal^158^ and epithelial to mesenchymal transition^159^. Some of these processes have already been described in endometriosis pathogenesis and they include Kras signalling^160,161^, MYC targets^162,163^, mTORC1 signalling^164–166^, PI3K AKT mTOR signalling^167–170^, TGF beta signalling^171–173^, interferon gamma^174–177^, and interferon alpha response^178,179^. In accordance with our data regarding microenvironment heterogeneity, certain pathways that are enriched in the stage III-IV phenotype are directly associated to M1-M2 macrophage polarization, and these pathways include TGF beta sinalling^180,181^, PI3K AKT mTOR signalling^151,182^, interferon gamma response^79^, adipogenesis, glycolysis and other metabolic reprograming pathways^183^.

Additionally, although they have not been identified as differently expressed based on the cut-offs used in this meta-analysis, some genes were involved simultaneously in several dysregulated pathways. Curiously, these genes are downregulated in eutopic endometrium from endometriosis women. *CDKN1B* encodes the protein p27kip1, a cyclin-dependent kinase inhibitor that prevents the activation of cyclin complexes and controls cell cycle progression to halt or slow cell division. It is a key regulator of endometriosis that exhibits potential utility in the diagnosis and treatment of this disease^184^. *SOD1* is pivotal for reactive oxygen species release during oxidative stress, and its expression is decreased in high stress environments. Peritoneal fluid from women with endometriosis can significantly reduce the expression of this gene and can contribute worsening reduction in oocyte quality^185^. Low levels of other genes within the endometrium may also be indicative of a hostile environment. These include *NOLC1*, which is fundamental for the biogenesis of nucleolar channel system in postovulation human endometrium^186^ and acts as a regulator of the acute phase response to alpha1-acid glycoprotein^187^, dihydrolipoamide dehydrogenase (*DLD*), which encodes a protein targeted by autoantibodies in women with endometrial cancer^188^. *H2AFZ*, which is involved in the cellular response to estradiol stimulus^189^, and *MTHFD2*, which was identified to be upregulated in ectopic endometria from women with endometriosis^190^. The role of these genes in the context of endometriosis remains unknown; however, given these punctual references to them, their potential roles in the pathogenesis of endometriosis requires further study.

### Strengths and limitations

The most significant advantage of our meta-analysis was that we analysed a selection of “real” healthy patients and a significant number of samples from the same microarray platform, and we classified these samples according to menstrual phase. Despite this advantage, this study did possess some limitations. First, despite the ability of Affymetrix platforms to provide higher correlations between gene expression profiles than can be obtained through RNA-Sequencing, the latter technique has been demonstrated to be superior in detecting low abundance transcripts, differentiating biologically critical isoforms, and allowing for the identification of genetic variants. This method also possesses a broader dynamic range than that of microarrays^191^. Second, removing batch effects is useful and necessary, although it may sometimes disturb downstream analysis by minimizing real biological difference among the experimental conditions^192^. Third, the paucity of clinical information such as detailed symptoms and lesion depth limit the ability to identify more associations between genotypes and phenotypes. Fourth, we recognize that numerous methods currently available to assess heterogeneity in the tissue microenvironment, however, we believe xCell is one of the most widely used and most robust tools based on the currently available data within the literature. Recently, a study systematically analysed the capacity and limitations of multiple transcriptome-based cell-type quantification methods^78^. According to this evaluation, xCell exhibits correlation indexes for predicting macrophages and NK cells of 0.96 and 0.88, respectively, which is superior to those provided by CIBERSORT. Additionally, significant biological and technical biases in regard to the in silico quantification of cell proportions are present when using matrices such as those used by CIBERSORT for deconvolution^193^. Finally, despite our belief that these pathways that were identified by the GSEA using MSigDB are in close agreement with the currently available literature, it must be noted that sometimes highly heterogenous findings are generated from the use of different and even similar enrichment analysis tools^193–196^. In fact, numerous methodological challenges must be overcome in the future. Even so, our study reinforces the importance of a periodic meta-analysis of “omics“studies.

## Conclusions

Our findings highlight that the cell subtypes present within the eutopic endometrium microenvironment, especially immune cell profiles, are different between samples obtained from women with stage I-II and stage III-IV endometriosis, and these differences are independent of the hormonal milieu. Specifically, a pro-inflammatory profile predominates in stage I-II endometriosis, andM1-M2 polarization into the eutopic endometrium may be crucial for progression of this disease. In contrast, the higher prevalence of NKT cells in eutopic endometria from women with endometriosis, independently of cycle phase or staging suggests a sustained stress and/or damage of the eutopic endometrium. Additionally, DEGs commonly expressed in earlier stages may indicate a response to local aggression. The results of this meta-analysis highlight the important challenges in treating this disease and provide opportunities for future research.

Beyond this, our meta-analysis also highlights at least 4 crucial points. Specifically, 1) it is possible to predict significant differences in tissue microenvironment of the samples by investigating the whole transcriptome using the microarray method and by reinforcing the key role of the immune system in the pathophysiology of the disease, 2) detailed phenotypic characterization of the patients diagnosed with endometriosis is fundamental for providing unbiased interpretations, and 3) the identification of DEGs with potential biological biomarker roles in this disease is possible, but these biomarkers may vary according to the fold change criterion and the menstrual cycle phase analysed, as few DEGs werecommonly identified in all phases of the menstrual cycle and almost all of them were associated with immunomodulation. Finally, 4) it is possible to identify enriched pathways and present them in a non-redundant way using the hallmark database to highlight those that are commonly involved in immunesurveillance, epithelial mesenchymal transition, stem cell processes, and macrophage polarization. Additionally, we recommend that some points should be necessarily reported when studying high dimensional genomic data in eutopic endometria owing theirpotential role as confounding variables. These points include the selection criteria of “real” controls, a precise definition of the menstrual phase, achieving a correct staging of the disease, and a description of the microenvironment that accounts for heterogeneity.

## Supplementary information

Dataset 1A.

Dataset 1B.

Dataset 2A.

Dataset 2B.

Dataset 2C.

Dataset 2D.

Dataset 2E.

Dataset 2F.

Dataset 3A.

Dataset 3B.

Dataset 3C.

Dataset 3D.

Dataset 3E.

Dataset 3F.

## Data Availability

The datasets used and analysed during the current study are available in GEO repository:GSE4888: Talbi S, Hamilton AE, Vo KC, Tulac S, Overgaard MT, Dosiou C, *et al*. Molecular phenotyping of human endometrium distinguishes menstrual cycle phases and underlying biological processes in normo-ovulatory women. Endocrinology 2006;147(3):1097–121. GSE6364: Burney RO, Talbi S, Hamilton AE, Vo KC, Nyegaard M, Nezhat CR, *et al*. Gene expression analysis of endometrium reveals progesterone resistance and candidate susceptibility genes in women with endometriosis. Endocrinology 2007;148(8):3814–26. GSE7305: Hever A, Roth RB, Hevezi P, Marin ME, Acosta JA, Acosta H, *et al*. Human endometriosis is associated with plasma cells and overexpression of B lymphocyte stimulator. Proc Natl Acad Sci 2007;104(30):12451–6. GSE51981: Tamaresis JS, Irwin JC, Goldfien GA, Rabban JT, Burney RO, Nezhat C, *et al*. Molecular Classification of Endometriosis and Disease Stage Using High-Dimensional Genomic Data. Endocrinology 2014;155(12):4986–99.
